# Correlates of caregiver well-being: The National Study of Caregivers

**DOI:** 10.3389/fpubh.2022.1059164

**Published:** 2023-01-10

**Authors:** Lydia C. Parr, Thelma J. Mielenz

**Affiliations:** Department of Epidemiology, Mailman School of Public Health, Columbia University Irving Medical Center, New York, NY, United States

**Keywords:** positive aspects of caregiving, NSOC, NHATS, Medicare, race

## Abstract

**Background:**

The literature demonstrates an association between aspects of caregiving and support with caregiver burden and differences by race. Our objective was to examine correlates of caregiver wellbeing, and if the effect is moderated by race.

**Methods:**

The National Study of Caregiving (NSOC) is a survey of unpaid and familial caregivers affiliated with participants in the National Health and Aging Trends Study, a nationally representative survey of Medicare beneficiaries. A total of 899 participants were examined cross-sectionally with logistic and multinomial logistic regression models to obtain adjusted odds ratios (aOR) and 95% confidence intervals (CI) for NSOC Round 3 (2017), stratified by race, to determine the association between aspects of caregiving and support variables with the two outcomes, three-level caregiving gains, and response to the statement “life has meaning and purpose.”

**Results:**

Among black caregivers with no family or friends to help, there were lower gains compared to very high gains (aOR: 2.82, 95% CI: 1.18, 6.77). Black and white caregivers who endorsed lower ratings regarding being appreciated by the care recipient had lower gains for “life has meaning and purpose” (aOR: 2.46, 95% CI: 1.00, 6.02; aOR: 1.65, 95% CI: 1.06, 2.56). Black caregivers with lower ratings regarding being appreciated had lower gains compared to very high gains (aOR: 5.04, 95% CI: 1.48, 17.17). White caregivers endorsing lower ratings to the same question had lower gains compared to very high gains (aOR: 3.27, 95% CI: 1.77, 6.04), and those with more help had lower gains (aOR: 0.81, 95% CI: 0.70, 0.93).

**Conclusion:**

The relationship between various correlates and positive aspects of caregiving is moderated by black and white races. Further study on the impact of aspects of caregiving and support networks for caregivers may shed light on factors contributing to racial differences and areas for intervention.

## 1. Introduction

With the increasing aging population of the United States, the population of unpaid and familial caregivers has also increased (1–3). Caregiver wellbeing, including the perception of purpose in life, is associated with better mental and physical health as well as decreased mortality (4). Prior studies found that caregiver wellbeing and burden are moderated by race/ethnicity for measures such as care burden, psychological wellbeing, and self-rated health, as well as varying use of support such as respite services based on race (5, 6). Additional research found evidence that black caregivers have higher levels of caregiving intensity such as assistance with activities of daily living (ADLs) as well as time spent on caregiving, and quality of life was most drastically impacted by caregiving intensity for female caregivers (7). The literature suggests negative emotional burden associated with high-intensity care and positive emotions related to caregiving vary by race and age, levels of social burden vary relating to caregiving intensity and caregiver's age, and high-intensity caregiving was associated with varying measures of quality of life for those of different racial groups, genders, ages, and incomes (3, 8–10). Other studies found associations between outlook, purpose in life, and positive caregiving outcomes for caregivers of older adults (4, 11, 12), indicating that both relationship and perception of the caregiving role may influence caregiver wellbeing. Demographic and personal-level factors, as well as domains aspects of caregiving, support environment, and duration of caregiving, all seem to contribute to caregiver wellbeing (5–8).

This study identifies key correlates of wellbeing and potential moderation regarding domains of caregiving and wellbeing. Most literature on unpaid caregiving for older adults focuses on the burden of care, and positive experiences and impacts on caregivers warrant attention as well (1, 13–16). This type of research may allow for public health policy and messaging regarding caregiving, which considers personal-level, socioeconomic, and political factors relating to caregiving outcomes rather than centering policy and messaging around negative aspects of caregiving. Such policy and messaging would be strengthened by an understanding of the effect social determinants related to race/ethnicity have on caregiver wellbeing. Examining what contributes to positive wellbeing outcomes may help better support caregivers, offer a balanced perception of caregiving, and make strides to improve wellbeing outcomes as demographic shifts continue to necessitate informal caregiving.

This study aims (1) to determine the correlates of caregiver wellbeing through analysis of the impact of the domains duration of care, aspects of caregiving, and support environment on caregiver wellbeing and (2) to study the wellbeing of caregivers based on both personal-level factors such as race and gender as well as domains of caregiving duration of care, aspects of caregiving, and support environment.

## 2. Methods

### 2.1. Data source and study design

Data were obtained from the National Study of Caregiving (NSOC) for the calendar year 2017 for this cross-sectional analysis. The NSOC is a nationally representative survey of family and other unpaid caregivers for older persons in the USA, and it has been conducted three times in conjunction with the National Health and Aging Trends Study (NHATS), which samples Medicare enrollees aged 65 and older (17).

### 2.2. Study population

For round 3, which represented caregivers initially interviewed in 2015 and re-interviewed in 2017, there were 2,361 caregivers interviewed. This study utilized 899 participants from the interview conducted in 2017 who did not have missing values for variables in the analyses. Caregivers are eligible if identified by an NHATS participant; assist with at least one of a list of mobility, self-care, household, and other activities; and are related to the NHATS participant (regardless of whether or not they are paid) or are unrelated and unpaid (17).

### 2.3. Variable classification

#### 2.3.1. Dependent variables

The primary outcomes are purpose in life and caregiving gains, which are derived from five variables in the NSOC.

The measure of caregiving gains is derived by summing four variables with four-level Likert responses that ask caregivers if their caregiving situation made them more confident about their abilities, taught them to deal with difficult situations, brought them closer to the care recipient, or had given them the satisfaction that the care recipient is well cared for (4).

The measure of purpose in life is derived from an NSOC question that asks caregivers to respond to the statement “My life has meaning and purpose” on a Likert scale from one to four (4, 12, 16).

#### 2.3.2. Independent variables

##### 2.3.2.1. Exposures

The potential exposures included in this analysis are as follows: duration of care measured in weeks of assistance and hours per week of care; aspects of caregiving such as relationship quality, perception of caregiving, living arrangement with the care receiver, relationship with the care receiver, and type of care provided such as assistance with ADLs and instrumental ADLs; support environments such as the caregivers' social network size and social participation as well as any social support with caregiving such as participation in support groups, training, or financial help.

##### 2.3.2.2. Covariates

Covariates for the model include the gender of the caregiver, coded dichotomously as male or female. The caregiver's age in years is continuous. Education level is categorized as less than high school, high school, and associate or beyond. In addition, one covariate will represent a number of chronic conditions (multiple morbidities) of the caregiver; this will be derived from dichotomous questions regarding if the caregiver has ever had a heart attack, heart disease, high blood pressure, arthritis, osteoporosis, diabetes, lung disease, cancer, difficulty seeing, difficulty hearing, chronic pain, breathing problems, limited strength in limbs, or fatigue. The number of comorbidities will be divided into the following categories: none, one, two, or three, and four or more (17, 18).

### 2.4. Statistical analysis

Associations between measures of caregiving gains and purpose in life with each potential exposure will be examined using the chi-square test. Linearity was assessed with age and ordinal variables as well as collinearity to identify any variables that were correlated. Due to collinearity with the hours help daily variable, how often help with chores, shopping, getting around the home, and help with personal care were omitted from the aspects of caregiving models. Whether the caregiver had attended a support group was omitted from the support model due to collinearity. Due to small cell sizes, certain categories were collapsed. For caregiving gains, the three lower strata were collapsed into one, including moderate, low, and very low gains. For “Life has meaning and purpose,” the responses “Agree somewhat” and “Disagree” were collapsed into one category. Other relative and non-relative relationships to the care recipient were collapsed into one category, with immediate family or spouse being the other category. Responses “A little” and “Not at all” to questions regarding the caregiving enjoying being with the care recipient, feeling that the care recipient appreciates them, and the frequency the caregiver speaks to the care recipient's medical provider were collapsed into one category for the analysis due to small cell sizes.

Nominal logistic regression and logistic regression analyses accounting for sampling weights will be performed to obtain adjusted odds ratios with 95% confidence intervals. We will also perform a Hosmer–Lemeshow test for goodness of fit of the logistic regression models to determine whether the model is adequate or whether there are correlates with a significant lack of fit. Variables included in this investigation will reflect hypotheses regarding the correlates of caregiver wellbeing, and analyses will be stratified by both race and gender, which were both seen to affect the modifiers of caregiving outcomes in prior studies (6–8). All analyses will be performed in StataBE v17.

## 3. Results

### 3.1. Sample characteristics

Table 1 shows unweighted counts and column proportions of caregiving and demographic variables by black and white races. For the “Life has meaning and purpose” variable, 91.08% of black caregivers endorsed “Agree Strongly,” while for white caregivers, a lesser proportion, 80.79%, endorsed “Agree Strongly.” For the “Caregiving gains” variable, 58.36% of black caregivers scored very high, while 30.32% of white caregivers scored very high. Black caregivers were slightly younger on average compared to white caregivers (56.29 vs. 61.55 years), and a greater proportion was female than white caregivers (77.7% vs. 57.9%). A smaller proportion of black caregivers were immediate family or spouse to the care recipient compared to white caregivers (85.25% vs. 91.9%), a greater proportion enjoyed being with the care recipient (91.08% vs. 77.94%), and a greater proportion reported speaking to the care recipient's medical provider “A Lot” (66.17% vs. 42.22%).

**Table 1:** Unweighted demographic characteristics.

	**Overall**		**Black**		**White**	

	***N***	**(%)**	***N***	**(%)**	***N***	**(%)**
**Caregiving gains**						
Very high	348	38.71	157	58.36	191	30.32
High	138	15.35	47	17.47	91	14.44
Moderate	245	27.25	44	16.36	201	31.9
Low	73	8.12	11	4.09	62	9.84
Very low	95	10.57	10	3.72	85	13.49
**Life has meaning and purpose**						
Agree strongly	754	83.87	245	91.08	509	80.79
Agree somewhat	117	13.01	14	5.2	103	16.35
Disagree	28	3.11	10	3.72	103	16.35
**Hours help per day**						
One	182	20.24	32	11.9	150	24.81
Two	188	20.91	46	17.1	142	22.54
Three	148	16.46	43	15.99	105	16.67
Four	91	10.12	29	10.78	62	9.84
Five to eight	181	20.13	70	26.02	111	17.62
More than nine	109	12.12	49	18.22	60	9.53
**Enjoy being with care recipient**						
A lot	736	81.87	245	91.08	491	77.94
Some	134	14.91	17	6.32	117	18.57
Little/not at all	29	3.23	7	2.6	22	3.49
**Care recipient appreciates you**						
A lot	758	84.32	235	87.36	523	83.02
Some	105	11.68	24	8.92	81	12.86
Little/not at all	36	4	10	3.72	26	4.13
**Relationship**						
Family or spouse	811	90.21	232	86.25	579	91.9
Other relative	64	7.12	29	10.78	35	5.56
Non-relative	24	2.67	8	2.97	16	2.54
**How often help with chores**						
Every day	324	36.04	125	46.47	199	31.59
Most days	131	14.57	61	22.68	70	11.11
Some days	207	23.03	54	20.07	153	24.29
Rarely	104	11.57	16	5.95	88	13.97
Never	133	14.79	13	4.83	120	19.05
**How often help with shopping**						
Every day	126	14.02	49	18.22	77	12.22
Most days	233	25.92	98	36.43	135	21.43
Some days	367	40.82	99	36.8	268	42.54
Rarely	104	11.57	12	4.46	92	14.6
Never	69	7.68	11	4.09	58	9.21
**How often speak to medical provider**						
A lot	444	49.39	178	66.17	266	42.22
Somewhat	261	29.03	52	19.33	209	33.17
A little	144	16.02	29	10.78	115	18.25
Not at all	50	5.56	10	3.72	40	6.35
**How often help getting around home**						
Every day	144	16.02	64	23.79	80	12.7
Most days	98	10.9	38	14.13	60	9.52
Some days	292	32.48	74	27.51	218	34.6
Rarely	171	19.02	47	17.47	124	19.68
Never	194	21.58	46	17.1	148	23.49
**How often help personal care**						
Every day	160	17.8	76	28.25	84	13.33
Most days	83	9.23	36	13.38	47	7.46
Some days	168	18.69	53	19.7	115	18.25
Rarely	173	19.24	54	20.07	119	18.89
Never	315	35.04	50	18.59	265	42.06
**Manage medical tasks**						
Yes	117	13.01	61	22.68	56	8.89
No	782	86.99	208	77.32	574	91.11
**Family and friends to talk to**						
Yes	804	89.43	241	89.59	563	89.37
No	95	10.57	28	10.41	67	10.63
**Family and friends help with caregiving**						
Yes	652	72.53	210	78.07	442	70.16
No	247	27.47	59	21.93	188	29.84
**Attended support group**						
Yes	43	4.78	16	5.95	27	4.29
No	856	95.22	253	94.05	603	95.71
**Received training**						
Yes	92	10.23	45	16.73	47	7.46
No	807	89.77	224	83.27	583	92.54
**Received financial help**						
Yes	165	18.35	76	28.25	89	14.13
No	734	81.65	193	71.75	541	85.87
Male	223	24.81	60	22.3	163	25.87
Female	676	75.19	209	77.7	467	74.13
Age	899	59.974 (20,93)	269	56.290 (21, 86)	630	61.548 (20, 93)
**Education**						
Less than high school	67	7.45	30	11.15	37	5.87
High school	424	47.16	143	53.16	281	44.6
Associate's or beyond	408	45.38	96	35.69	312	49.52
**Work for pay last week?**						
Yes	365	40.6	114	42.38	251	39.84
No	304	33.82	94	34.94	210	33.33
Retired/don't work	230	25.58	61	22.68	169	26.83
**Comorbid conditions**						
Four or more	385	42.83	102	37.92	283	44.92
Two or three	256	28.48	76	28.25	180	28.57
One	123	13.68	49	18.22	74	11.75
None	135	15.02	42	15.61	93	14.76

### 3.2. Support models

Multivariate regression results of the support models, which included correlates with family or friends to talk to, family or friends help caregiving, received financial help, and received caregiver training, found significant correlates among black caregivers. The “Life has meaning and purpose” and support model did not converge after accounting for sampling weights, and the caregiver training was completely determined for the unweighted model. Among black caregivers who had no friends or family to talk to, the unweighted analysis indicated there was 3.43 times the odds of endorsing “Somewhat Agree” or “Disagree” rather than “Strongly Agree” to the statement “Life has meaning and purpose” compared to those who did have family or friends to talk to, adjusting for education, number of comorbidities, caregiver age, and caregiver gender [adjusted odds ratio (aOR): 3.43, 95% confidence interval (CI): 1.08, 10.91]. The covariate received caregiver training predicted black caregivers' responses to “Life has meaning and purpose” perfectly. For the “Life has meaning and purpose” and support covariates model, family or friends to help caregiving, having received financial help, and having received caregiver training were not significant for black caregivers in the unweighted model, and no correlates were significant for white caregivers in the weighted model (Table 2).

**Table 2:** Life has meaning and purpose and support.

	**Black** ***N*** =**269**		**White** ***N*** = **630**	

**Characteristic**	**Odds ratio**	**95% CI**	**Odds ratio**	**95% CI**
Family or friends to talk to	**3.43**	**1.08, 10.91**	1.32	0.63, 2.74
Family or friends to help caregiving	0.63	0.20, 2.01	0.94	0.56, 1.59
Received financial help	0.73	0.28, 1.90	0.80	0.45, 1.41
Received caregiving training	Completely determined	N/A	0.57	0.25, 1.29

The reference category is agree strongly. Models adjusted for education, number of comorbidities, caregiver age, and caregiver gender. This model for black caregivers is unweighted because the weighted model failed to converge. The model for white caregivers is weighted. Bold indicates aORs that were found to be significant.

In the weighted analyses, among black caregivers with no family or friends to help with caregiving, there was 2.82 times the odds of having moderate to very low gains rather than very high gains compared to those with family or friends to help caregiving, adjusting for education, number of comorbidities, caregiver age, and caregiver gender (aOR: 2.82, 95% CI: 1.17, 6.77). Among black caregivers who had not received caregiving training, there was 4.52 times the odds of having high rather than very high gains compared to those with family or friends to help with caregiving, adjusting for education, number of comorbidities, caregiver age, and caregiver gender (aOR: 4.52, 95% CI: 1.43, 14.25). For the caregiving gains and support model, having family or friends to talk to and having received financial help were not significant correlates of caregiving gains for black caregivers, and no correlates were significant for white caregivers (Table 3).

**Table 3:** Caregiver gains and support.

**Characteristic**	**Black** ***N*** = **269**		**White** ***N*** = **630**	

	**Odds ratio**	**95% CI**	**Odds ratio**	**95% CI**
**Very high gains**	1.00		1.00	
**High gains**				
Family or friends to talk to	0.45	0.09, 2.29	1.48	0.54, 4.07
Family or friends to help caregiving	1.96	0.72, 5.31	0.53	0.27, 1.04
Received financial help	1.45	0.63, 3.33	1.79	0.70, 4.53
Received caregiving training	**4.52**	**1.43, 14.25**	1.75	0.51, 5.97
**Moderate to very low gains**				
Family or friends to talk to	0.95	0.30, 2.94	2.14	0.96, 4.76
Family or friends to help caregiving	**2.82**	**1.18, 6.77**	0.70	0.42, 1.18
Received financial help	1.26	0.55, 2.90	1.36	0.75, 2.47
Received caregiving training	2.35	0.87, 6.36	1.08	0.49, 2.36

The reference category is very high gains. Models are both weighted and adjusted for education, number of comorbidities, caregiver age, and caregiver gender. Bold indicates aORs that were found to be significant.

### 3.3. Aspects of caregiving models

The aspect of caregiving models includes variables relating to whether the caregiver has helped with medical tasks, talks to medical providers, their relationship with the care recipient (immediate relative or spouse vs. other), if they believe the care recipient appreciates them, and the hours they help the care recipient daily. For the model aspects of caregiving and “Life has meaning and purpose,” among black caregivers, those who endorsed responses “Some,” “Little,” or “Not at all” to the statement that the care recipient appreciates them had 2.45 times the odds of endorsing “Agree somewhat” or “Disagree” rather than “Agree strongly” to the statement “Life has meaning and purpose” compared to those who said the care recipient appreciates them “A lot,” adjusting for education, number of comorbidities, caregiver age, and caregiver gender (aOR: 2.45, 95% CI: 1.00, 6.02).

Among white caregivers, those who endorsed responses “Some,” “Little,” or “Not at all” to the statement that the care recipient appreciates them had 1.65 times the odds of endorsing “Agree somewhat” or “Disagree” rather than “Agree strongly” to the statement “Life has meaning and purpose” compared to those who said the care recipient appreciated them “A lot,” adjusting for the same demographic covariates (aOR: 1.65, 95% CI: 1.06, 2.56). Across the aspects of caregiving and “Life has meaning and purpose” models, the caregiver helping with medical tasks, talking to medical providers, relationship with the care recipient, and hours spent helping daily were not significant correlates of response to the statement “Life has meaning and purpose.” Both models were weighted (Table 4).

**Table 4:** Life has meaning and purpose and aspects of caregiving.

**Characteristic**	**Black** ***N*** = **269**		**White** ***N*** = **630**	

	**Odds ratio**	**95% CI**	**Odds ratio**	**95% CI**
Help with medical tasks	1.10	0.28, 4.28	1.06	0.45, 2.49
Talk to medical providers	1.08	0.50, 2.33	1.23	0.89, 1.71
Relationship	0.68	0.13, 3.56	0.47	0.19, 1.15
Care recipient appreciates you	2.46	1.00, 6.02	**1.65**	**1.06 2.56**
Hours help daily	0.84	0.63, 1.13	0.95	0.82 1.10

The reference category is agree strongly. Models are both weighted and adjusted for education, number of comorbidities, caregiver age, and caregiver gender. Bold indicates aORs that were found to be significant.

For the models relating to aspects of caregiving and caregiving gains, among black caregivers who only “Somewhat,” “A little,” or “Not at all” spoke to medical providers, there was 2.15 times the odds of very low to moderate gains rather than very high gains compared to those who talked to medical providers “A lot” (aOR: 2.15, 95% CI: 1.32, 3.50). Among white caregivers who only “Somewhat,” “A little,” or “Not at all” spoke to medical providers, there was 1.65 times the odds of very low to moderate gains rather than very high gains compared to those who talked to medical providers “A lot” (aOR: 1.65, 95% CI: 1.19, 2.29).

Among black caregivers who endorsed “Some,” “Little,” or “Not at all” to the statement that the care recipient appreciates them, there was 5.04 times the odds of very low to moderate gains rather than very high gains compared to those who endorsed the care recipient appreciates them “A lot” (aOR: 5.04, 95% CI: 1.48, 17.17). Among white caregivers who endorsed “Some,” “Little,” or “Not at all” to the statement that the care recipient appreciates them, there was 3.27 times the odds of very low to moderate gains compared to very high gains compared to those who endorsed the care recipient appreciates them “A lot” (aOR: 3.27, 95% CI: 1.77, 6.04).

Among black caregivers helping the care recipient at a one-category increase of hours (one, two, three, four, five to eight, and greater than nine) per day, there was 0.72 times the odds of very low to moderate gains rather than very high gains compared to those who helped the care recipient for a lower time category (aOR: 0.72, 95% CI: 0.57, 0.93). Among white caregivers helping the care recipient at a one-category increase of hours per day, there was 0.81 times the odds of very low to moderate gains rather than very high gains compared to those who helped the care recipient for a lower time category (aOR: 0.81, 95% CI: 0.70, 0.93). Helping with medical tasks and relationship with care recipients were not significant correlates of caregiving gains for black or white caregivers. Both models were weighted (Table 5).

**Table 5:** Caregiver gains and aspects of caregiving.

**Characteristic**	**Black** ***N*** = **269**		**White** ***N*** = **630**	

	**Odds ratio**	**95% CI**	**Odds ratio**	**95% CI**
**Very high gains**	1.00		1.00	
**High gains**				
Help with medical tasks	1.68	0.61, 4.66	0.56	0.14, 2.26
Talk to medical providers	1.64	0.91, 2.97	0.93	0.62, 1.40
Relationship	0.62	0.19, 2.01	0.87	0.31, 2.49
Care recipient appreciates you	2.82	0.79, 10.01	1.09	0.48, 2.47
Hours help daily	0.96	0.77, 1.20	0.86	0.72, 1.03
**Moderate to very low gains**				
Help with medical tasks	0.73	0.28, 1.94	1.05	0.43, 2.61
Talk to medical providers	**2.15**	**1.32, 3.50**	**1.65**	**1.19, 2.29**
Relationship	0.37	0.12, 1.22	0.93	0.41, 2.09
Care recipient appreciates you	**5.04**	**1.48, 17.17**	**3.27**	**1.77, 6.04**
Hours help daily	**0.72**	**0.57 0.93**	**0.81**	**0.70, 0.93**

The reference category is Very High Gains. Models are both weighted and adjusted for education, number of comorbidities, caregiver age, and caregiver gender. Bold indicates aORs that were found to be significant.

## 4. Discussion

Many of the observed associations aligned with our expectations. In the caregiving gains and aspects of caregiving models, for both races, those who endorsed lower frequencies of helping with medical tasks were more likely to have moderate, low, and very low compared to very high caregiving gains. This relationship warrants further study.

The models showed differences by black and white races, which was expected. Previous research using NSOC data found differences in caregiver outcomes by race (5). For the support models, “Life has meaning and purpose” and caregiving gains were only significant among black caregivers. Having family and friends to talk to related to endorsing “Agree strongly” to the statement “Life has meaning and purpose,” and for black caregivers without family or friends to help with caregiving, there were elevated odds of moderate, low, and very low compared to very high caregiving gains. The ORs for “Care recipient appreciates you” were significant for both races but differed in the “Life has meaning and purpose” and aspects of the caregiving model. In the caregiving gains and aspects of the caregiving model, the magnitude of the ORs differed by race for talking to medical providers and care recipient appreciates you. The high compared to very high caregiving gains category was only significant for black caregivers, and relationship with care recipient and hours help daily were only significant among white caregivers.

Higher caregiving intensity was represented by the hours help daily variable, which was found to be significantly associated with lower categories of caregiving gains for white caregivers. The relationship between caregiving intensity and caregiver burden has been previously established (7, 8). Social support variables and the caregiver's outlook on caregiving were found to be associated with positive wellbeing outcomes in previous studies as well (4, 8). This study provides further evidence that social support and a positive perception of the relationship with the care recipient relate to caregiver wellbeing.

This study has a few limitations. The constructs of caregiver wellbeing have not been widely studied, and other positive outcomes may be more appropriate. Causal relationships cannot be determined from this cross-sectional analysis. For both predictor and outcome variables, categories had to be collapsed due to small strata, but the resulting categories were still meaningful. There was a high proportion of missing data, which may cause bias and a lack of precision, and there may be a lack of generalizability because only black and white races were included in the analysis, while the non-Hispanic Other and Hispanic categories were excluded due to small cell sizes.

This is a preliminary analysis, and a future study will utilize multiple time points of the NSOC for longitudinal analysis to see whether the relationship persists across three time points when the third wave of longitudinal data is available. Studies on the impact of aspects of caregiving and support networks for caregivers may shed light on factors contributing to racial differences. In addition, further study may identify any areas for intervention and causal explanation for some of the associations identified in this study.

## Data availability statement

Publicly available datasets were analyzed in this study. This data can be found here: National Study of Caregiving. Produced and distributed by www.nhats.org with funding from the National Institute on Aging [Grant numbers R01AG054004 (NSOC III) and R01AG062477 (NSOC IV)].

## Ethics statement

The studies involving human participants were reviewed and approved by Columbia University Research Compliance and Administration System IRB. The patients/participants provided their written informed consent to participate in this study.

## Author contributions

TM and LP were involved in conceptualizing data analysis, manuscript preparation, interpretation of data, and critical revisions of the manuscript. LP performed data analysis. TM contributed to and supervised data analysis. All authors contributed to the article and approved the submitted version.

## References

[1] Cohen SA Cook S Kelley L Sando T Bell AE. Psychosocial factors of caregiver burden in child caregivers: results from the new national study of caregiving. Health and Quality of Life Outcomes. (2015) 13:120–5. 10.1186/s12955-015-0317-2 26246132 PMC4527125

[2] Haley WE West CA Wadley VG Ford GR White FA Barrett JJ Roth DL. Psychological, social, and health impact of caregiving: a comparison of black and white dementia family caregivers and noncaregivers. Psychol Aging. (1995) 10:540–52. 10.1037/0882-7974.10.4.540 8749581

[3] Willert B Minnotte KL. Informal caregiving and strains: exploring the impacts of gender, race, and income. Appl Res Qual Life. (2021) 16:943–64. 10.1007/s11482-019-09786-1

[4] Polenick CA Sherman CW Birditt KS Zarit SH Kales HC. Purpose in life among family care partners managing dementia: links to caregiving gains. Gerontologist. (2019) 59:e424–32. 10.1093/geront/gny063 29873736 PMC6857691

[5] Moon HE Haley WE Rote SM Sears JS. Caregiver well-being and burden: variations by race/ethnicity and care recipient nativity status. Innov Aging. (2020) 4:igaa045. 10.1093/geroni/igaa045 33241124 PMC7679974

[6] Parker LJ Fabius CD. Racial differences in respite use among black and white caregivers for people living with dementia. J Aging Health. (2020) 32:1667–75. 10.1177/0898264320951379 32819177

[7] Cohen SA Cook SK Sando TA Brown MJ Longo DR. Socioeconomic and demographic disparities in caregiving intensity and quality of life in informal caregivers: a first look at the national study of caregiving. J Gerontol Nurs. (2017) 43:17–24. 10.3928/00989134-20170224-01 28253411

[8] Cook SK Snellings L Cohen S. A socioeconomic and demographic factors modify observed relationship between caregiving intensity and three dimensions of quality of life in informal adult children caregivers. Health Qual Life Outcomes. (2018) 16:169. 10.1186/s12955-018-0996-6 30157852 PMC6116379

[9] Skarupski KA McCann JJ Bienias JL Evans DA. Race differences in emotional adaptation of family caregivers. Aging Mental Health. (2009) 13:715–24. 10.1080/13607860902845582 19882410 PMC2868599

[10] Sorensen S Pinquart M. Racial and ethnic differences in the relationship of caregiving stressors, resources, and sociodemographic variables to caregiver depression and perceived physical health. Aging Mental Health. (2005) 9:482–95. 10.1080/13607860500142796 16024408

[11] Lawton MP Moss M Kleban MH Glicksman A Rovine M. A two-factor model of caregiving appraisal and psychological well-being. J Gerontol. (1991) 46:P181–P189. 10.1093/geronj/46.4.p181 2071844

[12] McKnight PE Kashdan TB. Purpose in life as a system that creates and sustains health and well-being: an integrative, testable theory. Rev General Psychol. (2009) 13:242–51. 10.1037/a0017152

[13] Cohen CA Colantonio A Vernich L. Positive aspects of caregiving: rounding out the caregiver experience. Int J Geriatr Psychiatry. (2002) 17:184–8. 10.1002/gps.561 11813283

[14] Mackenzie A Greenwood N. Positive experiences of caregiving in stroke: a systematic review. Disabil Rehabil. (2012) 34:1413–22. 10.3109/09638288.2011.650307 22309576

[15] Mollica MA Smith AW Kent EE. Caregiving tasks and unmet supportive care needs of family caregivers: a US population-based study. Patient Educ Counsel. (2020) 103:626–34. 10.1016/j.pec.2019.10.015 31704030

[16] Sánchez-Izquierdo M Prieto-Ursúa M Caperos JM. Positive aspects of family caregiving of dependent elderly. Educ Gerontol. (2015) 41:745–56. 10.1080/03601277.2015.1033227

[17] Freedman VA Skehan ME Hu M Wolff J Kasper JD. National Study of Caregiving I-III User Guide. Baltimore: Johns Hopkins Bloomberg School of Public Health (2019). Available online at: www.nhats.org (accessed December 28, 2022).

[18] Positive Aspects of Caregiving Team: Kick Off Meeting. (2021). Geriatric Assessment and Rehabilitation Program-International Longevity Centres Global Alliance Caregiving Project. Ottawa.

